# Renal Tubular Acidosis With High Anion Gap: A Report of Two Cases

**DOI:** 10.7759/cureus.117896

**Published:** 2026-10-14

**Authors:** Padala Ravi Kumar, Kapileswar Swain, Deepak K Dash, Debasish Patro, Kamalakhya Samantaraya

**Affiliations:** 1 Department of Endocrinology, Maharaja Krishna Chandra Gajapati Medical College and Hospital, Berhampur, IND

**Keywords:** distal renal tubular acidosis, high anion gap metabolic acidosis, hypokalemia, organic acids, rickets

## Abstract

We report two children, one aged three years and six months and another five years and seven months, who presented with growth failure and progressive skeletal deformities. On examination, they had short stature, frontal bossing, rachitic rosary, Harrison sulcus, pectus carinatum, widened wrists, and genu valgum, suggestive of rickets. The X-rays of the wrist joints showed typical features like metaphyseal cupping, fraying, splaying and widening of the growth plate. Further investigations revealed metabolic acidosis with a high anion gap and urine pH >5.5. There was hypokalemia, hypercalciuria, and phosphaturia in both patients, and one of the patients also had nephrocalcinosis, suggestive of distal renal tubular acidosis. The delta gap ratios in the two children were 0.8 and 0.57, respectively, suggestive of mixed high- and normal-anion-gap metabolic acidosis. The presence of high-anion-gap metabolic acidosis led to further investigations. The urinary organic acid profiling revealed elevated 2-deoxytetronic acid, phenylacetic acid, thioglycolic acid, palmitic acid, and malonic acid, suggestive of an additional organic acid metabolic disturbance. So, the final diagnosis of distal renal tubular acidosis with an additional organic acid metabolic disturbance was made and managed with alkali (sodium bicarbonate), potassium supplementation for correction of hypokalemia, levocarnitine, biotin, and vitamin B12 as metabolic supportive therapy. On subsequent follow-up at six months, there was clinical improvement, normalization of blood pH, and anion gap. These cases highlight the importance of evaluation for an additional organic acid metabolic disturbance in patients with renal tubular acidosis and high anion gap.

## Introduction

Renal tubular acidosis (RTA) comprises a group of disorders characterized by impaired renal handling of acid or bicarbonate, resulting in metabolic acidosis despite relatively preserved glomerular filtration [1,2]. Distal RTA (dRTA) results from impaired hydrogen ion (H⁺) secretion by the alpha-intercalated cells of the collecting ducts. The three principal defects implicated in dRTA involve the H⁺-ATPase, anion exchanger 1 (the bicarbonate-chloride exchanger), and H⁺-K⁺-ATPase pathways [1,2]. These defects typically result in hyperchloremic, normal-anion-gap metabolic acidosis, hypokalemia, hypercalciuria, and nephrocalcinosis. In children, dRTA may manifest as growth failure, rickets, and short stature [2]. The presence of high anion gap metabolic acidosis (HAGMA) in patients with RTA is unusual and rare. The possible causes of HAGMA, like lactic acidosis, ketoacidosis, renal failure, and drug exposure, should be investigated [3,4]. Here, we report two cases of dRTA presenting with HAGMA. After exclusion of common causes of HAGMA, increased urinary excretion of organic acids may account for the elevated anion gap (AG) observed in these patients.

## Case presentation

Case 1

A three-year-six-month-old female child was brought by her parents as she had poor gain in height and weight, along with swelling of both wrist and ankle joints and inability to stand without support over a period of one year. She was the second-order child of a non-consanguineous marriage and was born by normal vaginal delivery with a birth weight of 2.6 kg. The perinatal and postnatal periods were reportedly uneventful, and her social and motor milestones were appropriate for age. She was able to stand with support at 11 months but was unable to stand without support at the time of presentation. She had adequate exposure to sunlight but poor nutritional status. There was no history of graveluria, recurrent abdominal pain, steatorrhea, vomiting, diarrhea, dehydration, salt craving, hypoglycemia, seizures, tetany, periodic paralysis, or fractures. There was no similar illness in the family. She had received calcium and vitamin D supplementation for approximately one year without any significant clinical improvement.

On examination, her height was 85 cm (-3.45 SDS (Standard Deviation Score)), weight was 8.3 kg (-4.87 SDS), and body mass index (BMI) was 11.49 kg/m² (less than third percentile). Upper-to-lower segment ratio was 1.125, and head circumference was 47 cm (third to 10th percentile). Blood pressure was 94/52 mmHg (50th to 90th percentile). Head-to-toe examination revealed frontal bossing, dental caries, rachitic rosary, Harrison sulcus, pectus carinatum, widened wrists, and genu valgum (Figure 1A-C). Radiographs demonstrated irregular epiphyses, widened physes, metaphyseal cupping, fraying, and splaying, all of which were suggestive of rickets (Figure 1D).

**Figure 1:**
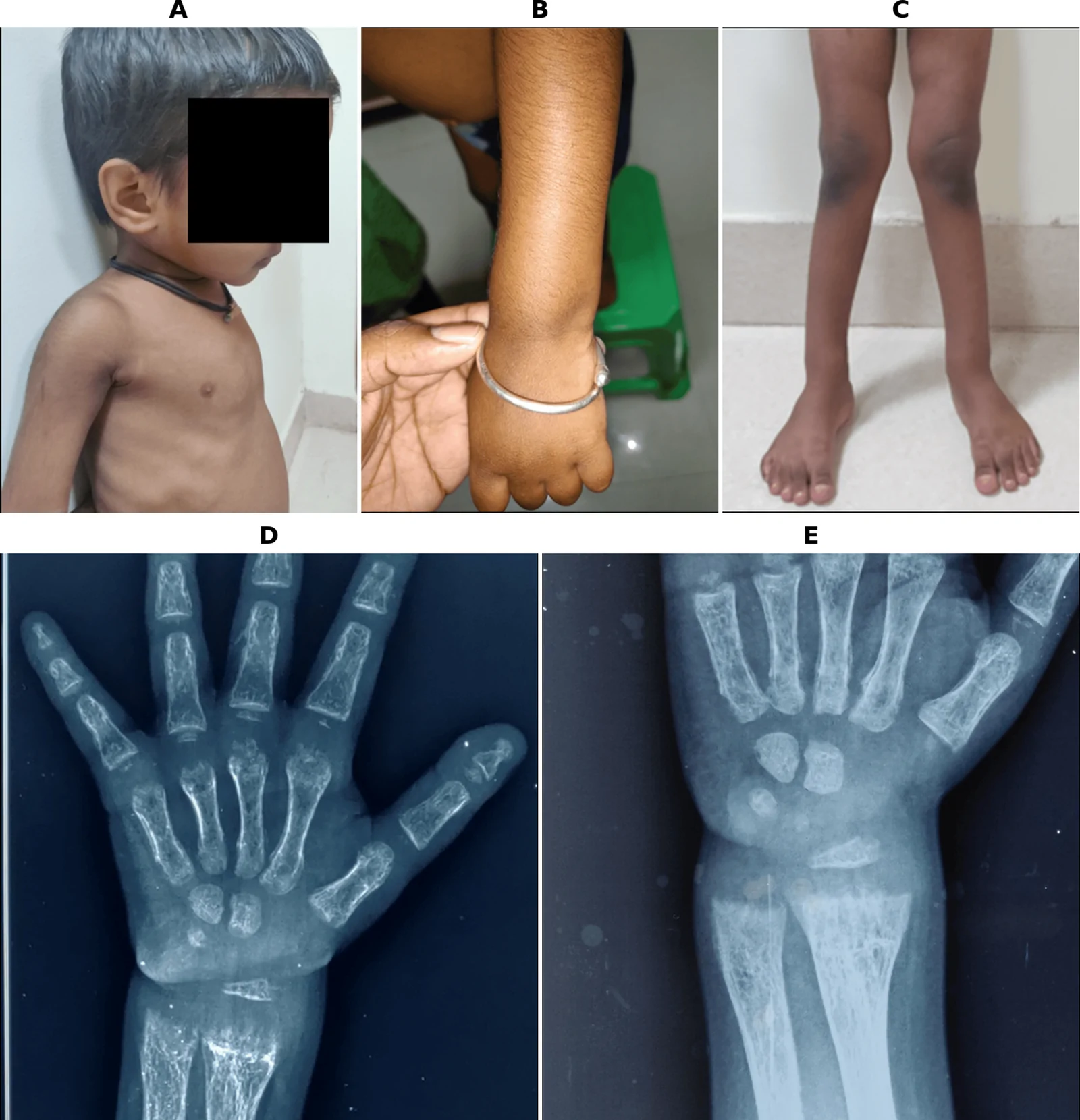
Clinical and X-ray findings in Case 1 (A) Rachitic rosary; (B) Widening of wrist joint; (C) Genu valgum deformity; (D) X-ray left hand (pre-treatment) showing metaphyseal cupping, fraying and splaying with epiphyseal thinning on presentation; (E) X-ray left hand (post-treatment) showing improvement of metaphyseal and epiphyseal rachitic findings. The patient's father consented to have the child's identity revealed, and a written and signed consent has been obtained.

Laboratory investigations revealed hypokalemia, hypophosphatemia, elevated alkaline phosphatase, normal serum calcium, and adequate 25-hydroxyvitamin D. Other hematological and biochemical parameters were within normal limits, as shown in Table 1. Arterial blood gas (ABG) analysis revealed a mean pH of 7.28 (normal range 7.35-7.45), bicarbonate of 14 mmol/L (normal range 22-26 mmol/L), partial pressure of carbon dioxide (PCO₂) of 27.6 mmHg (normal range 35-45 mmHg), and anion gap of 20 mmol/L (normal range 8-12 mmol/L), suggestive of HAGMA. The delta gap ratio was 0.8, consistent with a mixed high-anion-gap and normal-anion-gap metabolic acidosis. The delta gap ratio is calculated as:

$\frac{\Delta \mathrm{AG}}{\Delta \mathrm{HCO}_3^-} = \frac{\mathrm{AG} - 12}{24 - \mathrm{HCO}_3^-}$

Urinary evaluation revealed a urine pH of 8.0, 24-hour urinary calcium and phosphorus excretion showed hypercalciuria and phosphaturia, with low tubular reabsorption of phosphorus (TRP) and tubular maximum reabsorption of phosphate per glomerular filtration rate (TmP/GFR). The combination of systemic metabolic acidosis and an inappropriately high urine pH, together with hypokalemia, hypercalciuria and phosphaturia, supported the diagnosis of dRTA. Because of the high anion gap component, common causes of HAGMA were evaluated. Serum beta-hydroxybutyrate and plasma lactate levels were within normal range (Table 1). Renal function tests were normal, and uremic acidosis was ruled out. Urinary organic acid screening revealed elevated 2-deoxytetronic acid (22.14%; cutoff <12.88%), phenylacetic acid (7.52%; cutoff <0.62%), thioglycolic acid (0.90%; cutoff <0.10%), and palmitic acid (45.80%; cutoff <23.34%), providing a possible explanation for observed HAGMA in this patient (Table 2). Ultrasonography (USG) of the abdomen revealed bilateral medullary nephrocalcinosis (Figure 2). The combination of clinical, radiological, and biochemical investigations led to a final diagnosis of dRTA with co-existing organic acid metabolic disturbance.

**Table 1:** Hematological, Biochemical, Hormonal, ABG and Urinary Investigations in Cases 1 and 2 ^α^Age-specific reference range for Case 1; ^β^age-specific reference range for Case 2. TSH: Thyroid-stimulating hormone, Free T4: free thyroxine, PTH: parathyroid hormone, ABG: arterial blood gas, pH: potential of hydrogen, HCO₃⁻: bicarbonate, PCO₂: partial pressure of carbon dioxide, AG: anion gap, TRP: tubular reabsorption of phosphate, TmP/GFR: tubular maximum reabsorption of phosphate per glomerular filtration rate.

Investigations	Case 1	Case 2	Reference range
Hemoglobin (g/dL)	13.9	11.3	11-16
Serum sodium (mEq/L)	142	144	135-145
Serum potassium (mEq/L)	2.6	2.8	3.5-5.2
Serum calcium (mg/dL)	10.4	10.2	9-11
Serum phosphate (mg/dL)	2.69	1.99	3.25-6.04^α^, 3.097-5.57^β^
Alkaline phosphatase (U/L)	1681	1143	156-369
25-Hydroxyvitamin D (ng/mL)	66.4	81.08	30-100
Serum magnesium (mg/dL)	2.2	2	1.7-2.2
Serum creatinine (mg/dL)	0.4	0.4	0.4-1.1
TSH (mIU/L)	1.85	1.93	0.27-4.2
Free T4 (ng/dL)	0.96	0.99	0.83-1.43
Intact PTH (pg/mL)	17.65	21.02	11-59
ABG pH	7.28	7.27	7.35-7.45
ABG HCO_3_^-^ (mmol/L)	14	12.1	22-26
ABG PCO_2_ (mmHg)	27.6	25.15	35-45
Anion gap (mmol/L)	20	18.9	8-12
Serum beta-hydroxybutyrate (mmol/L)	0.4	0.3	<0.6
Plasma lactate (mg/dl)	12.6	6.2	4.5-19.8
Urine pH	8.0	7.0	-
24-Hour urine volume (L)	1.8	1.2	-
24-hour urinary calcium (mg/kg/day)	11.92	4.4	<4
Urine calcium/Creatinine ratio (mg/mg)	0.712	0.38	<0.41^α^, <0.30^β^
24-Hour urinary phosphate (mg)	468	154	—
TRP	0.50	0.706	0.80-0.95
TmP/GFR (mg/dL)	1.34	1.405	3.25-5.5^α^, 3-5.1^β^

**Figure 2:**
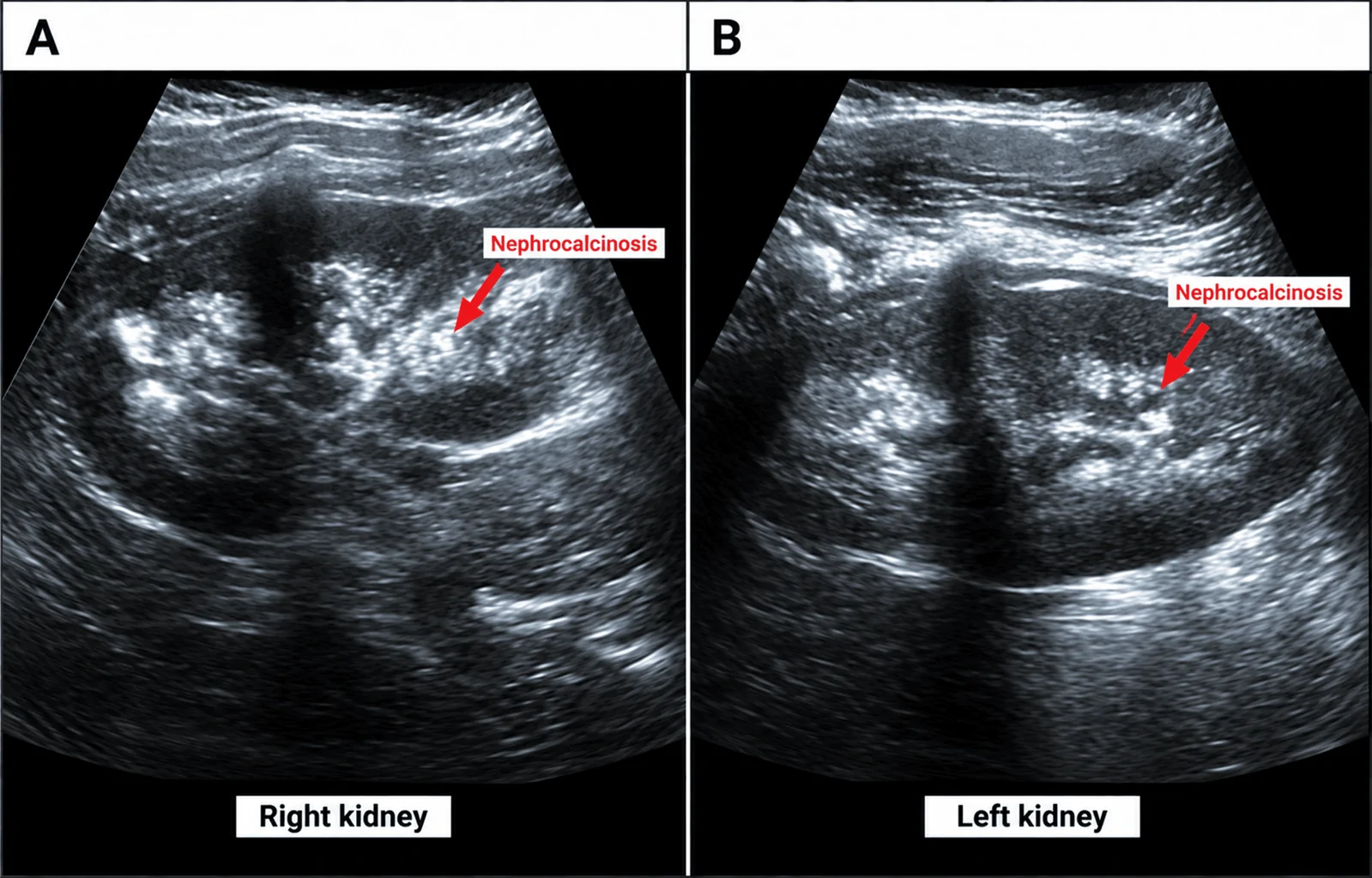
Ultrasonography abdomen of Case 1 (A) Right kidney showing increased echogenicity of the renal medullary region, with red arrow indicating the area of nephrocalcinosis. (B) Left kidney showing similar increased medullary echogenicity, with red arrow indicating nephrocalcinosis.

Case 2

A five-year-and-seven-month-old female child presented with poor gain in height and weight for three and a half years, and swelling of both wrists and ankles with difficulty in standing for one year. She was the first-order child of a non-consanguineous marriage and was born by normal vaginal delivery with a birth weight of 2.85 kg. The perinatal and postnatal periods were uneventful. There was a history of fracture involving the lower end of the left femur and bending of the right upper arm following trivial trauma six months back. She was adequately exposed to sunlight. There was no history of graveluria, recurrent abdominal pain, steatorrhea, vomiting, diarrhea, dehydration, salt craving, hypoglycemia, seizures, tetany, periodic paralysis, or any similar illness in the family.

On examination, her height was 82 cm (-5.86 SDS), weight was 9 kg (-5.50 SDS), and BMI was 13.38 kg/m² (10th to 25th percentile). Upper-to-lower segment ratio was 1.1, and head circumference was 48 cm (fifth to 15th percentile). Blood pressure was 92/54 mmHg (50th to 90th percentile). Head-to-toe examination revealed dental caries, rachitic rosary, Harrison sulcus, pectus carinatum, widened wrists, and kyphoscoliosis (Figure 3A-C). Radiographs showed metaphyseal cupping, fraying, and splaying with irregular epiphyses, consistent with rickets (Figure 3D).

**Figure 3:**
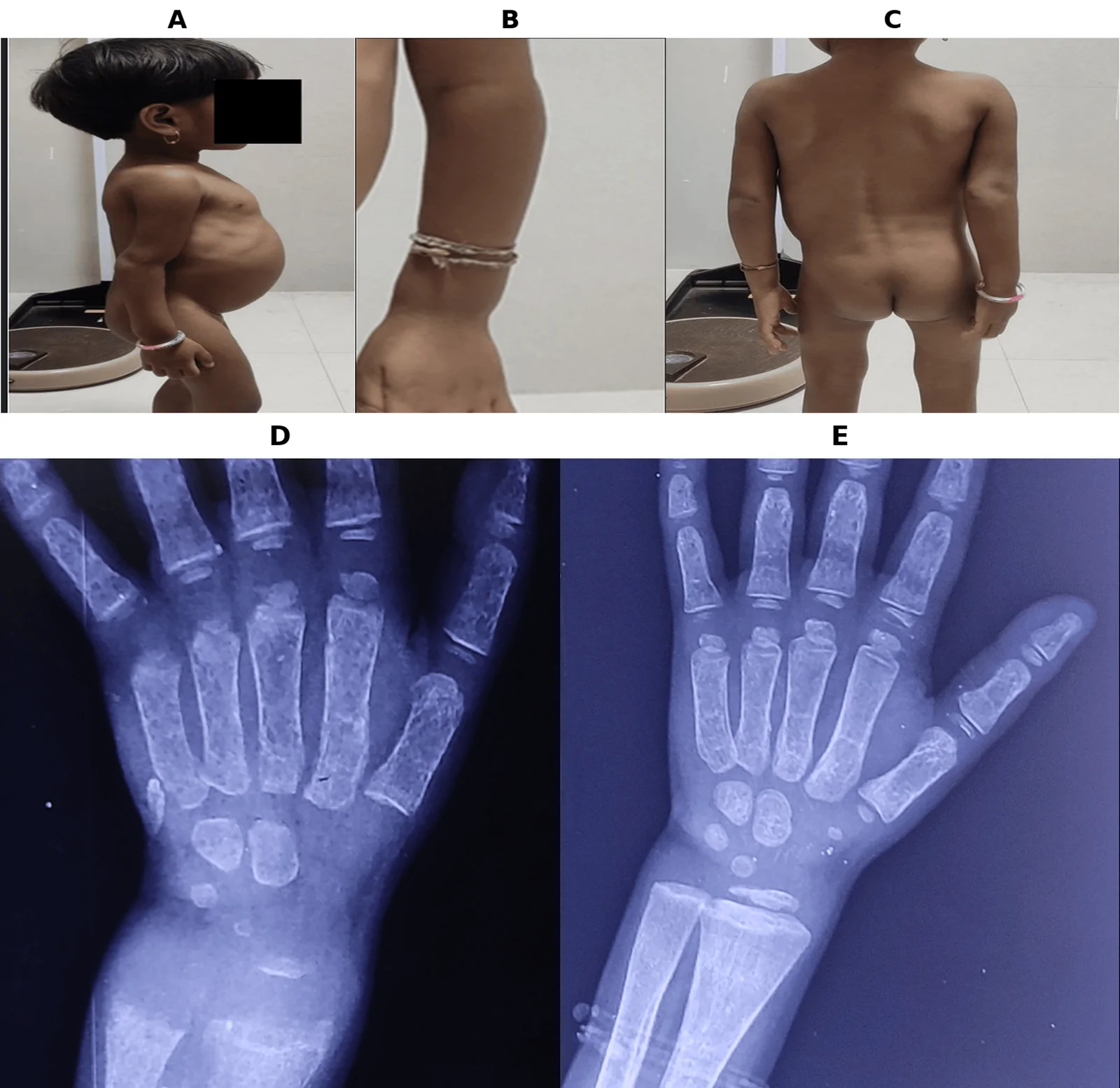
Clinical and radiographic findings in Case 2 (A) Rachitic rosary, Harrison sulcus, protuberant abdomen; (B) Widening of wrist joint; (C) Kyphoscoliosis; (D) X-ray left hand (pre-treatment) showing metaphyseal cupping, fraying and splaying with epiphyseal thinning on presentation; (E) X-ray left hand (post-treatment) showing improvement of metaphyseal and epiphyseal rachitic findings. The patient's father consented to have the child's identity revealed, and a written and signed consent was obtained.

Laboratory investigations demonstrated hypokalemia and hypophosphatemia with markedly elevated alkaline phosphatase, while serum calcium and 25-hydroxyvitamin D levels were within the normal range; the remaining hematological and biochemical parameters are summarized in Table 1. ABG analysis showed a pH of 7.27 (normal range 7.35-7.45), bicarbonate of 12.1 mmol/L (normal range 22-26 mmol/L), PCO₂ of 25.15 mmHg (normal range 35-45 mmHg), and an anion gap of 18.9 mmol/L (normal range 8-12 mmol/L), suggestive of HAGMA. A delta gap ratio of 0.57 indicated a mixed high-anion-gap and normal-anion-gap metabolic acidosis.

Urinary studies showed a urine pH of 7.0, with hypercalciuria and phosphaturia, along with reduced TRP and low TmP/GFR. The presence of systemic metabolic acidosis with an inappropriately elevated urine pH, along with hypokalemia, hypercalciuria, and phosphaturia, supported the diagnosis of dRTA. In view of the high-anion-gap component, the causes of HAGMA were assessed. Serum beta-hydroxybutyrate and plasma lactate were within normal limits, and preserved renal function made uremic acidosis unlikely (Table 1). Urinary organic acid screening showed elevated malonic acid (14.13%, cutoff <0.50%), phenylacetic acid (5.43%, cutoff <0.62%), 3-(3-hydroxyphenyl)-3-hydroxypropionic acid (38.15%, cutoff <4.99%), and thioglycolic acid (0.71%, cutoff <0.10%), providing biochemical evidence of an additional organic acid metabolic disturbance (Table 2). The clinical, radiological and biochemical findings supported a diagnosis of dRTA with co-existing organic acid metabolic disturbance.

**Table 2:** Urinary Organic Acid Profiling in Cases 1 and 2

Organic acids	Case 1	Case 2	Lab cutoff
2-Deoxytetronic acid (%)	22.14	-	<12.88
Phenylacetic acid (%)	7.52	5.43	<0.62
Thioglycolic acid (%)	0.90	0.71	<0.10
Palmitic acid (%)	45.80	-	<23.34
Malonic acid (%)	-	14.13	<0.50
3-(3-Hydroxyphenyl)-3-hydroxypropionic acid (%)	-	38.15	<4.99

Both children were treated with alkali therapy using sodium bicarbonate at 3 mEq/kg/day and potassium supplementation at 1 mmol/kg/day [1]. Levocarnitine 100 mg/kg/day, biotin 10 mg/day, and vitamin B12 1000 mcg/day were also administered as metabolic supportive therapy [5]. At six months of follow-up, both children demonstrated clinical improvement of rachitic features, radiological resolution of metaphyseal and epiphyseal changes (Figure 1E, 2E), with normalization of blood pH, anion gap (AG), bicarbonate, serum potassium and phosphates, as shown in Table 3.

**Table 3:** Follow-up ABG and Biochemical Parameters in Cases 1 and 2 ABG: Arterial blood gas; pH: Potential of hydrogen,  HCO₃⁻: bicarbonate.

Parameter	Case 1		Case 2	
	Baseline	At 6 Months Follow-up	Baseline	At 6 Months Follow-up
pH	7.28	7.35	7.27	7.36
HCO₃⁻ (mmol/L)	14	22	12.1	22.57
Anion Gap (mmol/L)	20	11	18.9	9.7
Serum potassium (mEq/L)	2.6	3.8	2.8	4.1
Serum phosphate (mg/dL)	2.69	3.75	1.99	4

## Discussion

These two cases highlight the importance of detailed work-up to arrive at a correct diagnosis in RTA. RTA usually presents with normal-anion-gap metabolic acidosis with hypokalemia, hypercalciuria, and nephrocalcinosis in dRTA [1,2]. The delta gap ratio, which is a ratio of delta change in anion gap divided by delta change in bicarbonate, helps in further work-up in such cases [3,6]. The delta gap ratio between 0.4 and 0.8, as was the case in these two cases, indicates mixed high- and normal-anion-gap metabolic acidosis. The next step in the work-up includes conditions that can present with HAGMA, like lactic acidosis, uremic acidosis, diabetic ketoacidosis, drug poisoning, and organic acidemia, should be investigated [3,4]. In our cases, lactic acidosis, ketoacidosis, and uremic acidosis were excluded by relevant investigations, and there was no history of any drug poisoning.

Organic acidemias are a heterogeneous group of inherited disorders of intermediary metabolism caused by defects in enzymes, transport proteins, or required cofactors involved in the catabolism of amino acids, carbohydrates, and lipids, particularly branched-chain amino acids like leucine, isoleucine, and valine, with a reported incidence of one in 3000 live births [4,7]. The metabolic block results in the accumulation of organic acids and related toxic intermediates in blood and tissues, with increased urinary excretion. Most clinically significant disorders present during catabolic stress in the neonatal period or infancy, although childhood or adulthood presentation can also occur. Clinical manifestations include poor feeding, vomiting, failure to thrive, hypotonia, lethargy, and recurrent episodes of metabolic acidosis [4]. Diagnosis is based on the clinical and biochemical phenotype and urinary organic acid analysis, usually by gas chromatography-mass spectrometry [7,8].

Organic acidemias can produce HAGMA because accumulated organic acids dissociate into hydrogen ions and their corresponding organic anions. The released hydrogen ions are buffered by bicarbonate, resulting in a fall in serum bicarbonate, while the negatively charged organic acid conjugates remain in the extracellular fluid as unmeasured anions and thereby increase the anion gap [3,4]. The common causes for organic acidemia include methylmalonic acidemia, propionic acidemia and isovaleric acidemia [4,7]. Urinary organic acid profiling in both cases demonstrated several abnormal metabolites, including 2-deoxytetronic acid, phenylacetic acid, thioglycolic acid, palmitic acid, malonic acid, and 3-(3-hydroxyphenyl)-3-hydroxypropionic acid, suggestive of an underlying disturbance of organic acid metabolism. The urinary organic acid profiles therefore provide a plausible biochemical explanation for the additional high-anion-gap component. The elevated urinary excretion of organic acids may be secondary to an underlying condition or may represent a non-specific finding. Management in these children should address both components of metabolic disturbances. The correction of acidosis and hypokalemia with sodium bicarbonate and potassium replacement, respectively, and organic acid metabolic disturbance with levocarnitine, biotin and vitamin B12 are effective strategies for the management of such cases.

This case report has certain limitations. First, urinary ammonium excretion, urine anion gap, and formal urinary acidification testing were not performed, and therefore the diagnosis of dRTA was based on the characteristic clinical and biochemical phenotype. Second, although urinary organic acid profiling demonstrated multiple abnormalities, confirmatory genetic evaluation, and molecular testing were not performed.

## Conclusions

Distal renal tubular acidosis typically presents with normal-anion-gap metabolic acidosis, hypokalemia, hypercalciuria, and high urine pH. In patients with suspected distal renal tubular acidosis who present with high-anion-gap metabolic acidosis, other causes of high anion gap metabolic acidosis, like lactic acidosis, ketoacidosis, renal failure, and conditions leading to organic acidosis, should be evaluated. Early diagnosis and targeted treatment can lead to significant clinical improvement and may prevent long-term skeletal and renal complications.
